# Supplementary material for: Mode-phase-difference photothermal spectroscopy for gas detection with an anti-resonant hollow-core optical fiber
Source: Nat Commun. 2020 Feb 12;11:847. doi: 10.1038/s41467-020-14707-0 (PMC7015925; doi:10.1038/s41467-020-14707-0)
Supplement: Supplementary file 1 — Supplementary Information [file 41467_2020_14707_MOESM1_ESM.pdf]

Supplementary Information

Mode-phase-difference photothermal spectroscopy for gas detection with an anti-resonant hollow-core optical fiber

Zhao et al.

Supplementary Note 1: Mode property of the SR-AR-HCF

The modal properties of the SR-AR-HCF were studied numerically. The geometry model is illustrated in Supplementary Fig. 1a, and the parameters used are based on the scanning electron spectroscopy (SEM) of the SR-AR-HCF used in our experiments. The fiber supports two groups of modes: the LP_{01}-like mode and the LP_{11}-like mode that includes the LP_{11a} and LP_{11b} modes. The calculated mode fields of the LP_{01} mode with two orthogonal polarizations at 1550 nm are shown in Supplementary Fig. 1b. The mode fields of the LP_{11a} and LP_{11b} modes are shown in Supplementary Fig. 1c. The effective refractive index (RI) difference between LP_{01} and LP_{11} is ~1.2 $\times 10^{-3}$. The effective RI difference between LP_{11a} and LP_{11b} is ~7×10^{-5}, which prevents coupling between them and makes the orientations of the mode intensity profile robust against small external perturbation. The effective RI difference is ~ 1.4×10^{-6} between the orthogonal polarizations of the LP_{11a} (or LP_{11b}) and ~1.8×10^{-7} between the orthogonal polarizations of the LP_{01}.

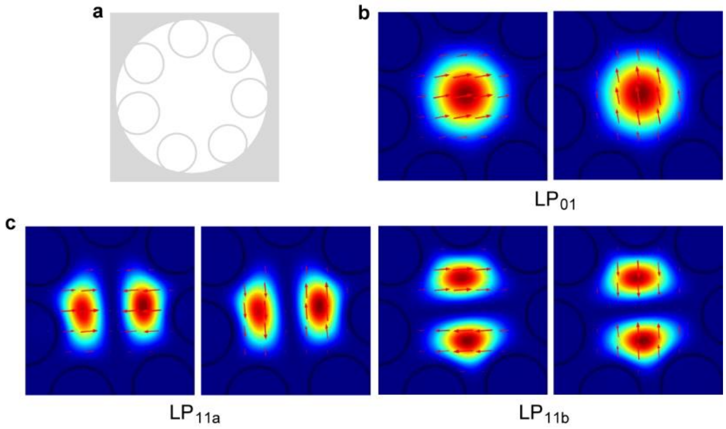

Supplementary Figure 1 Model of the SR-AR-HCF and the calculated mode fields at 1550 nm. a The geometry of the model. **b**, **c** Intensity profiles of (**b**) LP_{01} and (**c**) LP_{11} including LP_{11a} and LP_{11b} modes. The surface plots are the time averaged Poynting vector along z axis, and the arrow direction shows the direction of electric field.

Supplementary Note 2: Sensitivity of MPD to internal gas absorption

Although the SR-AR-HCF supports both LP_{11a} and LP_{11b} modes. Only one of them (e.g., LP_{11b}) is excited in the experiment. The normalized mode fields of the LP_{01} and the LP_{11} modes are labelled respectively as ψ_{01} and ψ_{11} for the pump and Ψ_{01} and Ψ_{11} for the probe, which satisfies $\langle\psi_i,\psi_i\rangle = 1$ and $\langle\Psi_i,\Psi_i\rangle = 1$ (i=01 or 11) with "$\langle\ \rangle$" represents the overlap integral over the fiber cross-section. The pump intensity variation along the HCF ($i.e.,z$-direction) may be expressed by

$$I_p = P_p\left[\eta\psi_{01}^2 + (1-\eta)\psi_{11}^2 + 2\sqrt{\eta(1-\eta)}\psi_{01}\psi_{11}cos\left(k_p\Delta nz\right)\right] \tag{2.1}$$

where P_p is the pump power, η is the fractional pump power in LP_{01} mode, k_p is the angular wavenumber of the pump and $\Delta n(= n_{01} - n_{11})$ is the effective RI difference between LP_{01} and LP_{11} modes.

Now we consider wavelength modulation spectroscopy in which the pump wavelength is modulated sinusoidally at frequency $\omega=2\pi f$ with a modulation index of m. The harmonic modulation of the pump wavelength results in a series of harmonic heat sources at multiple of the modulation frequency. In our experiment, we concern about the second harmonic (2f) heat source. For spectroscopic gas detection at room temperature, the gas absorption line-shape may be regarded as Lorentzian. The heat source at $2f$ may be expressed as[1]

$$\tilde{Q} = H_2 \alpha_0 C I_p e^{j2\omega t} = Q e^{j2\omega t} \tag{2.2}$$

where

$$H_2 = \frac{2}{m^2}\left(\frac{m^2+2}{\sqrt{m^2+1}} - 2\right) \tag{2.3}$$

is the second harmonic coefficient. α_0 is the peak absorption coefficient that equals to 1.051 cm^{-1} for the P(13) line of C_2H_2 at 296K and 1 atm, C is the gas concentration of C_2H_2 and 2ω is the angular frequency of the second harmonic. In our experiment, the amplitude of wavelength modulation is 2.2 times of the absorption linewidth (i.e., $m = 2.2$), giving the maximum value of $H_2 = 0.343$.

Under the assumption that absorption is weak and thermal conduction is the dominating heat dissipation process, heat conduction in the HCF may be studied by using

$$j\omega\rho C_p \tilde{T} - \kappa \nabla^2 \tilde{T} = \tilde{Q} \tag{2.4}$$

where ρ, C_p and κ are respectively the density, the heat capacity at constant pressure, and the thermal conductivity of the gas in the hollow-region or silica for the seven rings. $\tilde{T}$ is the harmonic perturbation of the temperature distribution. The inner wall of the outer silica cladding is regarded as the boundary with constant temperature. Since the gradient of heat source along the z-direction is much smaller than that in the radial directions, we may ignore the heat conduction in the z-direction and replace ∇^2 by the transverse operator ∇_T^2. Under this assumption and using Eqs. (2.1-2.2) and (2.4), the temperature field $\tilde{T}$ may be expressed as

$$\tilde{T} = T_1(x,y)e^{j2\omega t} + T_2(x,y)cos(k_p \Delta nz)e^{j2\omega t} \tag{2.5}$$

The temperature field varies periodically along the fiber with a spatial period equal to the mode beat length l_b~1.3 mm. Using the standard perturbation theory, the perturbation of mode index at the probe wavelength may be calculated by the overlap integral over the fiber cross-section [2, 3]

$$\Delta n_{01}(z,t) = \langle \Delta n(x,y,z), \Psi_{01}^2 \rangle \tag{2.6}$$

$$\Delta n_{11}(z,t) = \langle \Delta n(x,y,z), \Psi_{11}^2 \rangle \tag{2.7}$$

where

$$\Delta n(x,y,z) = -\frac{n_0 - 1}{T_0}\tilde{T} \tag{2.8}$$

is the perturbation of RI of the gas in the hollow-core, n_0 is the RI of the original gas without heating, and T_0 the ambient temperature. Here we have ignored the change in the RI of silica because the optical modes are almost completely in the air-core (less than 3×10^{-4} of power in silica for the SR-AR-HCF used in the experiment). The overall perturbation in the MPD between the two modes is then

$$\Delta\phi = k_s \int_0^L [\Delta n_{01}(z,t) - \Delta n_{11}(z,t)]dz \tag{2.9}$$

where k_s is the angular wavenumber of probe. From Eqs. (2.5-2.9) we could figure out that, over the integer multiple of the modal beam length, the second term on the right side of Eq. (2.5) would have negligible contribution to the overall change in the MPD. This term is accumulated (averaged) over many periods of mode beat length (more than 500 periods) and approaches zero since the PT induced change in the MPD over one beat length is very small. Hence, we may approximately the change in the MPD as

$$\Delta\phi = -\frac{k_s(n_0-1)Le^{j\omega t}}{T_0}\langle T_1(x,y), \Psi_{01}^2 - \Psi_{11}^2 \rangle \tag{2.10}$$

where $T_1(x,y)$ is the solution of

$$j\omega\rho C_p T_1(x,y) - \kappa\nabla_T^2 T_1(x,y) = H_2\alpha_0 C P_p[\eta\psi_{01}^2 + (1-\eta)\psi_{11}^2] \tag{2.11}$$

The modulation coefficient k^* is then determined as

$$k^*(\eta,\omega) \equiv \frac{|\Delta\phi|}{\alpha_0 C L P_p} = \frac{k_s(n_0-1)}{T_0}\langle \widehat{T}_1(x,y), \Psi_{01}^2 - \Psi_{11}^2 \rangle \tag{2.12}$$

where $\widehat{T}_1(x,y)$ is the solution of

$$j\omega\rho C_p \widehat{T}_1(x,y) - \kappa\nabla_T^2 \widehat{T}_1(x,y) = H_2[\eta\psi_{01}^2 + (1-\eta)\psi_{11}^2] \tag{2.13}$$

Eq. (2.13) was numerically solved with the parameters given in Supplementary Table 1. Supplementary Fig. 2 shows the modulation coefficient k^* as function of the heat modulation frequency. The value of k^* shows no significant change at low frequencies and approximately halved at ~58 kHz for argon (~67 kHz for nitrogen).

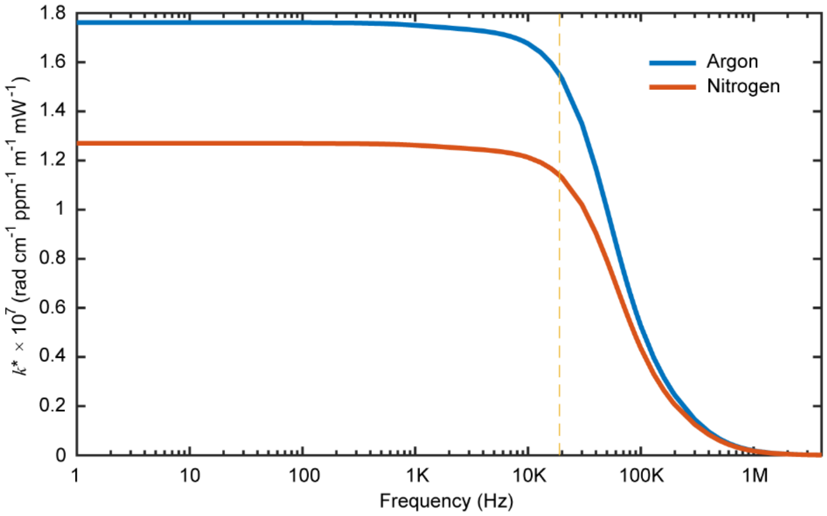

Supplementary Figure 2 Modulation coefficient k^* as function of heat-modulation frequency that is the second harmonic frequency (2*f*) of pump modulation. The results are obtained under the condition of ambient temperature and the pressure of 1.5 bar. The vertical dashed line indicated the operating frequency (2*f*) in our experiments.

For the parameters used in our experiments, i.e., η=96%, $2f$=19 kHz and probe wavelength λ=1550 nm, k^* is determined to be ~1.6×10^{-7} rad cm ppm^{-1} m^{-1} mW^{-1} and ~1.2×10^{-7} rad cm ppm^{-1} m^{-1} mW^{-1} with argon and nitrogen as the balance gas, respectively. We choose the pump modulation at f=9.5 kHz (i.e., heat-modulation $2f$=19 kHz) because it corresponds to that the k^* value decreases to ~ 88% of its maximum, which is obtained at low modulation frequency. In principle, we could operate at any frequency below or around 19 kHz without significantly compromising the PT differential phase modulation. However, the detection noise is of a $1/f$ dependence and hence we choose the pump wavelength modulation frequency to be 9.5 kHz, which corresponds to 19 kHz of the heat-modulation frequency (i.e., second harmonic of the pump modulation). Operating at this frequency simultaneously achieves large PT phase modulation as well as lower detection noise, maximizing the signal to noise ratio (SNR).

In our experiments (see Fig. 3), the pump and probe are lunched into the SR-AR-HCF from different ends, which means that the pump and the probe could be in the same (e.g., both are in LP_{11b}) or different LP_{11} (e.g., pump in LP_{11b} while probe in LP_{11a}) modes. To examine the effect of different combination of the pump and probe modes on the differential PT phase modulation, we numerically calculated the differential PT phase modulation coefficient for various pump and probe mode combinations for varying fractional pump power η in the LP_{01} mode. The results are shown

in Supplementary Fig. 3. According to the simulation results, for η>80% (which is the case for the two samples used in our experiments), the change of modulation coefficient k* is within 20% of the maximum value (i.e., the value for η=100%). This means that it is not a big issue whether the pump and the probe are launched into the same or different LP_{11} modes, as long as most of the pump power is in the LP_{01} mode. Supplementary Fig. 3 also shows that it is better to launch the pump and the probe into orthogonal LP_{11} mode (i.e., one in LP_{11a} and the other in LP_{11b}) because the dependence on η is much weaker. The best choice is of course to launch all the pump power into the LP_{01} mode (i.e., η=100%), where the k* value is always maximized and around 1.6x10^{-7} rad cm ppm^{-1}m^{-1}mW^{-1}. This could in principle be done by launching the pump into LP_{01} mode (via perfect alignment) and then using a long period grating inscribed along the SR-AR-HCF to resonantly couple part of the probe (not the pump) to LP_{11a} (or LP_{11b}) to form an in-line probe dual-mode interferometer while leaving the pump beam unaffected.

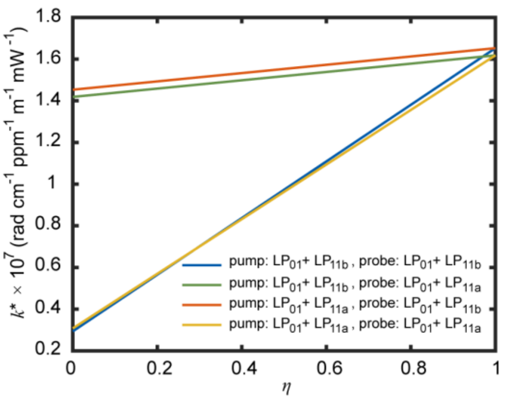

Supplementary Figure 3 Modulation coefficient k^* as function of η for different combinations of LP_{01} and LP_{11} modes of pump and probe beam. The results are obtained under the condition of ambient temperature and 1.5 bar gas pressure.

Supplementary Table 1. Parameters of materials at ambient temperature and pressure

Property	Silica	Polymer	Argon	Nitrogen
Density ρ [kg/m^3]	2203	1190	1.80	1.16
Thermal conductivity κ [W/(m·K)]	1.38	0.19	0.0174	0.0256
Heat capacity at constant pressure C_p [J/(kg*K)]	703	1420	520	1040
Refractive index n_0	1.45	1.54	1.000263	1.000284
Thermal expansion coefficient α [K^{-1}]	5.5×10^{-7}	1.8×10^{-4}	-	-
Thermo-optic coefficient dn/dT [K^{-1}]	9.5×10^{-6}	-1.3×10^{-4}	$-(n_0-1)/T_0$	$-(n_0-1)/T_0$
Young's modulus E [GPa]	73.1	0.035	-	-
Poisson's ratio ν	0.17	0.40	-	-

Supplementary Note 3: Sensitivity of MPD to external temperature and pressure

The sensitivity of the fundamental mode (i.e., LP_{01}) phase and the MPD to external temperature and pressure were studied via the model shown in Supplementary Fig. 4. Other parameters used in the simulation are listed in Supplementary Table 1. External temperature and pressure were treated as uniform perturbations to the HCF. Under the assumption that the perturbations are small, the phase change is linear to the amplitude of external perturbations.

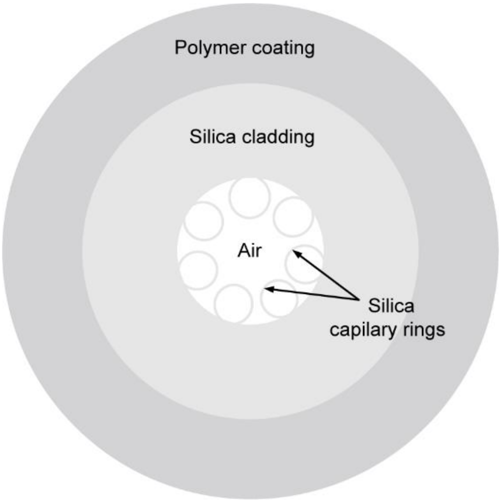

Supplementary Figure 4 Geometry model of the SR-AR-HCF for numerically calculating the phase sensitivity of LP_{01} mode and MPD to external perturbations. The SR-AR-HCF includes a ring-shaped polymer outer coating with thickness of ~80 μm, a ring-shaped silica cladding with outer diameter of ~190 μm and inner diameter of ~56 μm, 7 thin capillary rings with diameter of ~14 μm and thickness of ~370 nm. The inscribed air-core has a diameter of ~28 μm.

Firstly, we consider phase sensitivity to temperature change. Generally speaking, two kinds of effects need to be taken into account in the simulation if we ignore the elastic-optic effect induced by thermal expansion[4]. First, the RI change of material due to temperature change, or the thermo-optic effect. Second, the longitudinal and transverse thermal expansion. Take the phase sensitivity of LP_{01} mode to temperature as an example:

$$S_{01,X} = \frac{\Delta\phi_{01}}{\Delta X} = \frac{2\pi n_{01} L}{\lambda}\left(\frac{1}{n_{01}}\frac{\partial n_{01}}{\partial X} + \frac{1}{L}\frac{\partial L}{\partial X}\right) \tag{3.1}$$

where X refers to temperature here (or pressure which will be discussed later). The first term on the right-hand side (RHS) accounts for change of effective RI induced by the transverse thermal expansion and the thermo-optic effect. The second term on the RHS accounts for the longitudinal expansion of the fiber. The thermal expansion of the fiber could be calculated using the Hooke's Law which relates the strain tensor $\boldsymbol{\epsilon}$ and the stress tensor $\boldsymbol{\sigma}$:

$$\boldsymbol{\epsilon} = \boldsymbol{C}:\boldsymbol{\sigma} + \boldsymbol{\alpha}\Delta T \tag{3.2}$$

where $\boldsymbol{C}$ is the compliance tensor which is related to the Young's modulus E and the Poisson's ratio ν, and $\boldsymbol{\alpha}$ is the thermal expansion coefficient which is isotropic and only has the diagonal elements. The parameters used in the simulation are listed in Supplementary Table 1. The phase sensitivity to temperature is closely related to the thermal property of polymer since polymer used for fiber coating has a much large thermal expansion coefficient compared with silica. In cylindrical coordinates, only the diagonal elements of $\boldsymbol{\epsilon}$ are nonzero for isotropic material (i.e. silica). By following the procedure of Ref. [5], we calculated the longitudinal and transverse thermal expansion numerically using COMSOL Multiphysics. Then the change of effective RI of optical modes is calculated numerically by considering both the thermo-optic effect and the transverse thermal expansion, using the parameters listed in Supplementary Table 1.

Secondly, we calculate the phase sensitivity to the external pressure perturbation by following the same procedure. The external pressure compresses the fiber and results in longitudinal elongation and transverse deformation. The boundary condition at the outer surface (normal vector $\hat{n}$) of polymer should satisfy

$$\boldsymbol{\sigma} \cdot \hat{\boldsymbol{n}} = -P\hat{\boldsymbol{n}} \tag{3.3}$$

where P is the relative external pressure with reference to 1 atm. With Hooke's Law, we could solve the strain field and then the displacement field numerically using COMSOL Multiphysics. The phase sensitivity to external pressure P was calculated using Eq. (3.1) with "*X*" representing pressure. The first and second terms on the RHS are referred to as the changes of effective RI induced by transverse deformation and longitudinal elongation, respectively.

The above analysis can also be applied to determine the sensitivity of the MPD to external perturbations:

$$S_{\mathrm{MPD},X} = \frac{\delta\phi}{\Delta X} = \frac{2\pi\Delta n L}{\lambda}\left(\frac{1}{\Delta n}\frac{\partial \Delta n}{\partial X} + \frac{1}{L}\frac{\partial L}{\partial X}\right) \tag{3.4}$$

where X refers to temperature T or pressure P. In the simulation, the contribution of elastic-optic effect to effective RI of optical modes is ignored because it is relatively small[6].

The ratio of the phase sensitivity of the LP_{01} mode to that of the MPD may be expressed as

$$\zeta = \frac{S_{01,X}}{S_{\mathrm{MPD},X}} \tag{3.5}$$

which represents the enhancement of system stability against external perturbations (e.g. temperature or pressure). Numerical simulation with COMSOL Multiphysics shows that the value of ζ is ~280 for temperature and ~170 for pressure for the SR-AR-HCF at 1550 nm. In other words, the sensitivity to temperature or pressure is a few hundred times smaller for the MPD than that for the phase of the LP_{01} mode.

Supplementary Note 4: Fabrication and characterization of the SR-AR-HCF gas cells

The SR-AR-HCF

The SR-AR-HCF was home-made by co-authors Shoufei Gao, Yingying Wang and Pu Wang. The fiber is not optimized for this application, but we found it supports two and only two LP modes (i.e., LP_{01} and LP_{11}) with stable mode patterns.

By varying the launch condition, i.e., lateral offset between SMF and SR-AR-HCF, we can excite LP_{01} mode in combination with one of the LP_{11} mode (i.e., either LP_{11a} or LP_{11b}). The two-lobe pattern of the excited LP_{11a} or LP_{11b} mode does not vary with launch condition, although the intensity level may change. No other higher-order mode was observed. And once the SMF/SR-AR-HCF joint is secured, perturbation on the SMF does not affect the lobe-orientation. Small perturbation on the SR-AR-HCF also does not affect the lobe-orientation. However, when a tight bend (of a few cm) is applied to the SR-AR-HCF, we observed conversion from LP_{11a} to LP_{11b} or vice versa. In our experiments, strong bend was avoided and hence the mode pattern does not change during the entire measurement process. This may be due to the considerably large effective RI difference between LP_{11a} and LP_{11b} modes (~$7\text{x}10^{-5}$) and the fact the bending doesn't introduce much modal-birefringence because most of the mode energy is in air and hence the mode birefringence is relatively insensitive to bending.

We hope to optimize the fiber in future so that it could support LP_{01} mode and only one of the LP_{11} modes (e.g., LP_{11a}). After completion of this work, we noticed a recently published paper[7], which states that stable guidance of preferred modes could be achieved relatively easily by altering the position of the silica capillary rings.

The 4.67-m-long SR-AR-HCF gas cell

Two gas cells are made with the SR-AR-HCF. The first one uses a 4.67-m-long HCF (Supplementary Fig. 5) with one end mechanically spliced to an 8° angled single mode fiber (SMF) with 1-2 μm gap between them for filling gas into the hollow-core. A lateral offset of a few μm is also introduced between the HCF and SMF, to excite the two probe modes (LP_{01} and LP_{11}) simultaneously. The coupling loss for the joint is 4-6 dB. The mechanical joint is AB glue sealed in a T-shaped glass tube with an inlet and an outlet for gas filling. The other end of the SR-AR-HCF is coupled into another SMF with 1~2 μm gap between them, the relative lateral positions of the SR-AR-HCF and the SMF ends are adjusted by use of 3-Axis stages to achieve good fringe contrast for the interference between the two modes. SR-AR-HCF was looped to an 8-turn coil with a diameter of ~18 cm and placed on an acrylic plate and fixed onto the plate by sellotape.

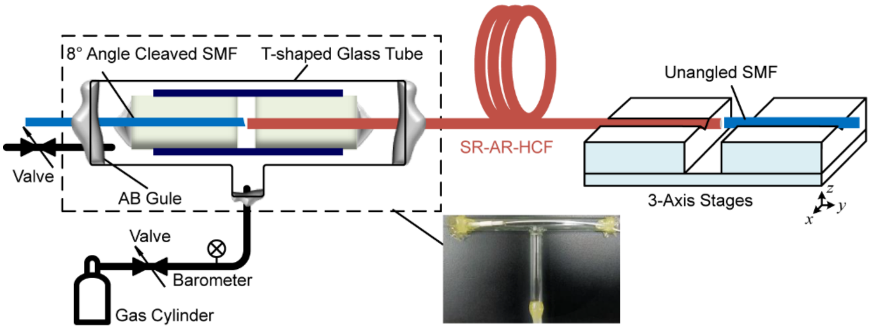

Supplementary Figure 5 Structure of the gas cell made with 4.67-m-long HCF. Insert: picture of the sealed SMF and SR-AR-HCF joint.

The transmission spectrum of the SR-AR-HCF gas cell is shown in Supplementary Fig. 6a, which is measured by the Optical Spectrum Analyzer with wavelength resolution of 10 pm. The mean insertion loss of the entire SR-AR-HCF gas cell is ~14dB. This relatively large loss is due to mode field mismatch and the lateral offset between SR-AR-HCF and SMFs. The average spacing or free spectral range (FSR) between adjacent interference fringe peaks is calculated to be ~435 pm. Supplementary Fig. 6b shows the fast Fourier transform (FFT) of the interference spectrum. There is only one peak at ~2.3 nm^{-1}, indicating only the LP_{11} mode is guided along with the LP_{01} mode and no other higher order modes exist. The power percentages of the LP_{11} and LP_{01} modes coupling into the output SMF are estimated to be 4% and 96% respectively.

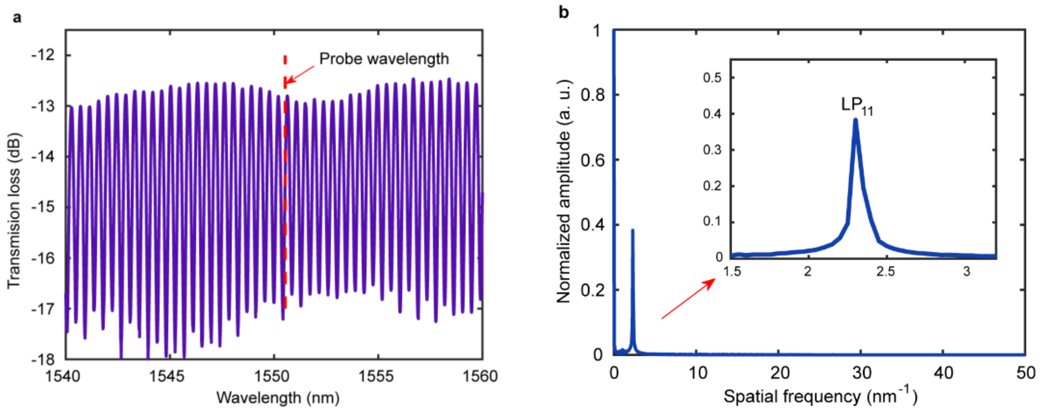

Supplementary Figure 6 Properties of the SR-AR-HCF gas cell. a Mode interference spectrum measured at the output of the gas cell. **b** FFT of the interference spectrum.

We could determine the differential effective mode RI through equation

$$\frac{2\pi\Delta nL}{\lambda_1} - \frac{2\pi\Delta nL}{\lambda_2} = 2\pi \quad (4.1)$$

that is

$$\Delta n = \frac{\lambda_1\lambda_2}{|\lambda_1 - \lambda_2|L} \approx \frac{\lambda^2}{\text{FSR}\cdot L} \quad (4.2)$$

where in our experiment L = 4.67 m, and $\lambda_1 \approx \lambda_2 \approx \lambda$ =1550 nm, so we get $\Delta n \approx 1.18 \times 10^{-3}$ which is very close to the calculated effective RI difference of 1.16×10^{-3} between LP_{01} and LP_{11b} modes, as mentioned in **Supplementary Note 1**. This cell was used to test the noise equivalent concentration (NEC) and the stability.

The 0.74-m-long SR-AR-HCF gas cell

For testing the dynamic range, we need to repeatedly fill the SR-AR-HCF with gas samples of different acetylene concentrations. To achieve fast gas-filling, we constructed a 0.74-m-long SR-AR-HCF gas cell with multiple microchannels along the HCF, which are made by use of an 800-nm femtosecond laser, as shown in Supplementary Fig. 7a. The 0.74-m-long HCF gas cell was mechanically spliced to SMFs in the input and the output ends with lateral offsets to form an all-fiber mode interferometer. The joint loss from SMF to SR-AR-HCF is relatively small, but the loss from SR-AR-HCF to SMF could go beyond 15 dB, which is influenced by lateral offset. SR-AR-HCF was kept straight and fixed onto a 1-m-long narrow aluminum plate by sellotape. A total of 34 microchannels were fabricated along the HCF and the separations between the microchannels range from ~2 cm to ~5 cm. The HCF gas cell with microchannels was placed inside a 1-m-long and 25-mm-diameter tubular gas chamber and gas filling into the hollow-core was achieved via these microchannels. Supplementary Fig. 7b shows the cross-section of the HCF with a microchannel. Supplementary Figs. 7c and 7d show respectively the transmission loss spectrum and average loss (over 1510-1560nm) for different number of drilled microchannels. Average loss is obtained from the DC value of FFT of transmission loss spectrum. The average loss per channel is ~0.013 dB. This cell was used to test the dynamic range and response time.

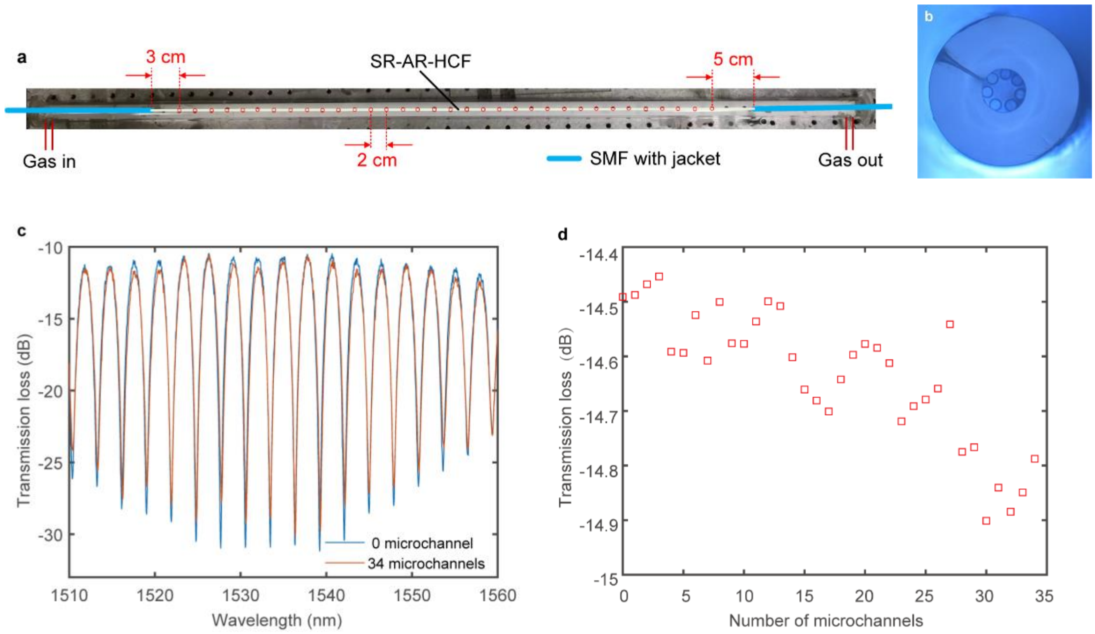

Supplementary Figure 7 Structure and properties of the 0.74-m-long SR-AR-HCF gas cell. a Picture of the

HCF gas cell. The HCF is fixed on an aluminum bar and placed inside a 1-m-long and 25-mm-diameter tubular gas chamber. 34 microchannels were drilled along the HCF, and the locations of the microchannels are indicted as red circles. **b** Cross-sectional image of a typical microchannel. **c** Transmission spectrum of the gas cell measured when increasing number of microchannels drilled along the HCF. **d** Loss (averaged from 1510 to 1560 nm) measured with increasing number of microchannels.

Supplementary Note 5: Experimental details

Test of lower detection limit and long-term stability

We tested the NEC and long-term stability by pressuring gas into the 4.67-long SR-AR-HCF with pressure level of ~1.95 bar at the input end of the SR-AR-HCF while output end open to atmospheric pressure. We used calibrated gas in a pressurized gas cylinder with known acetylene concentration for these experiments, and a complete filling of gas into the whole length of SR-AR-HCF took about 4 min. Without pressuring, it could take over 3 days based on calculation according to reference [8]. The measurement was conducted after the target gas fills the entire length of the SR-AR-HCF and reaches a steady flow state. During the experiment, the measured gas kept flowing through the SR-AR-HCF and the gas pressure distribution along the fiber may be expressed as[9]

$$P(z) = \sqrt{P_A^2 + \frac{z}{L}(P_B^2 - P_A^2)} \tag{5.1}$$

with

$$\begin{cases} P_A = 1.95 \text{ bar} \\ P_B = 1 \text{ bar} \\ L = 4.67 \text{ m} \end{cases}$$

where L is the length of fiber, $P_{A,B}$ the gas pressure at the ends of SR-AR-HCF (input end A and output end B), and z the position with respect to input end A. The average pressure over the entire length of SR-AR-HCF may be calculated as

$$P_{avr} = 1/L\int_0^L P(z)dz \approx 1.5\ bar \tag{5.2}$$

For gas pressure from 1 to a few bars, the shape of the P(13) line of acetylene may be approximated as Lorentzian[10]. However, the center of the line would shift slightly by ~ -209 MHz and linewidth changes from ~4.7 to ~12.9 GHz, when the pressure varies from 1 to 1.95 bar.

The experimental setup is shown in Fig. 3. We used commercial laser current (Thorlabs, LDC205C) and temperature (Thorlabs, TED200C) controllers in combination with the internal signal generator of lock-in amplifier (Stanford Research Systems, SR830) to drive the pump source (Distributed-feedback laser, Eudyna-FLD5F15CX) and perform modulation and an EDFA (Amonics, AEDFA-EX) for power amplification. The probe source is an external-cavity diode laser (Agilent 81600B), and signal is detected by a photodetector (Nirvana-2017). In the present work, we simply tuned the probe wavelength, at the beginning of each experiment, to a quadrature point of dual-mode interference fringe. The system was very stable, and the operating point remained almost unchanged over the entire measurement period in the lab environment. We didn't use any feedback controller to stabilize the operating point. Instead, we simultaneously detected the $2f$ signal (Fig. 5a) from the lock-in and the slow-varying (we call it DC) component from the photodetector (not shown in Fig. 3). The DC component was denoised by using Daubechies wavelet (db5) at level

6 and the denoised DC signal (V_{DC}) is shown as the blue curve in Supplementary Fig. 8, while the peak-to-peak(p-p) value of the $2f$ signal is shown as the red square dot. The p-p value shows a similar trend as the DC signal, which would include the effect of intensity fluctuation of probe as well as the drift of the operating point. Then, $2f$ signal was DC-compensated by multiplying it with a compensation factor ($\overline{V_{DC}}/V_{DC}$, $\overline{V_{DC}}$ is the average value of V_{DC} over three hours), and the p-p value of the DC-compensated $2f$ signal is shown in Fig. 5c. For real time applications, stable operation may be achieved by servo controlling the probe wavelength to track the fringe quadrature.

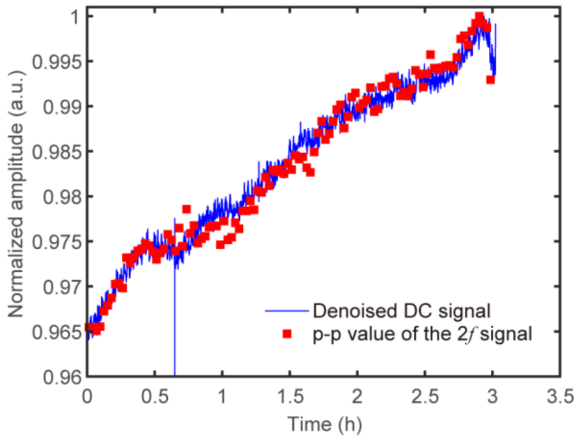

Supplementary Figure 8 The p-p value of the $2f$ signal and the denoised DC signal.

The experimental results are shown in Fig. 4 and Fig. 5 in the main paper. The 1σ NEC for a lock-in time constant of 1s is 68 ppt. It should be stated that lock-in detection with 1s lock-in time constant (with 18 dB/oct roll-off) corresponds an equivalent detection bandwidth of ~0.094 Hz (see the handbook of the SR830 lock-in). If we use the ideal low-pass filter model with sharp cutoff frequency, this corresponds to an equivalent integration time of ~10s (instead of 1s). From the Allan-Werle plot (i.e., Fig. 4c), 10s averaging time corresponds to NEC of ~43 ppt, which is not far away from the 1σ NEC of ~68 ppt obtained from the lock-in measurement. According to reference [11], for white phase noise, the equivalent degrees of freedom of Allan-Werle variance would be smaller for a longer integration time resulting in narrower confidence interval; the error then becomes relatively larger and the oscillation more serious.

Test of dynamic range and response time

Instead of the 4.67-m-long SR-AR-HCF, we used the 0.74-m-long sample to test dynamic range and the response time of the system. The reasons for not using the longer SR-AR-HCF sample is that we need to repeat the experiment many times with the SR-AR-HCF filled with different gas concentrations, which were done by mixing the calibrated gas with known acetylene concentration with high-purity nitrogen by use of mass flow controllers in our lab. The gas mixtures are at ~1 bar and we do not have the facility to pressurize the mixed gas sample to a higher-pressure level while maintaining high precision in concentration and pressure level. Theoretically we could drill many microchannels along the 4.67-meter-long SR-AR-HCF and fill the gases through these microchannels via diffusion. However, it is practically difficult for us to do so at the moment. The femtosecond laser micro-machining system we used could only fabricate high quality filling-microchannels on a straight hollow-core fiber that is fixed to a plate before the start of microchannel-drilling. Previously, we made hundreds of microchannels along a single hollow-core photonic

crystal fiber (HC-PCF) by fixing the HC-PCF on a plate with multiple straight sections with small bends between them. However, the state-or-the-art SR-AR-HCF cannot be bent to small diameters (the bending loss is ~8 dB/m for bend diameter smaller than 4 cm[12]), hence the arrangement with multiple straight sections with a small bend in between (to connect the straight sections) cannot be achieved. In our experiment, we coiled the 4.67-meter-long SR-AR-HCF into loops of~18 cm in diameter and secured it on the plate with sellotape. We found it is extremely difficult to fabricate multiple high-quality microchannels on the curved SR-AR-HCF using the present micro-machining setup.

However, we were able to drill multiple microchannels on a shorter length of straight SR-AR-HCF (i.e., ~0.74 m) that was fixed to an aluminum plate, the SR-AR-HCF was mechanically spliced to SMFs at both ends, which allows on-line monitoring the transmission loss during the fabrication of the microchannels. For this shorter SR-AR-HCF sample, the hollow-core can be filled from the filling-microchannels repeatedly, at around atmospheric pressure, with gas of different concentrations in a short time without the need to pressurize the gas.

The results of dynamic range and response time tests are shown in Figs. 6 and 7 in the main paper. The NEC for the 0.74-m-long SR-AR-HCF was also tested by filling the calibrated 1ppm acetylene in argon into the SR-AR-HCF at the pressure of 1.5 bar. The 1σ NEC obtained from the lock-in detection with 1s time constant and 18 dB/oct roll-off (corresponding to detection bandwidth of 0.094Hz) is 2 ppb. Allan-Werle variance analysis is also conducted with noise data over a period of 2 hours, and the results are shown in Supplementary Fig. 9, the Allan-Werle deviation reduces with averaging time and the 1σ NEC goes down to ~500 ppt for ~100 s averaging time. The system could average over a longer time, e.g., >1000 s, showing the system is stable over a longer term.

Comparing with the 4.67-m-long sample, the ratio of NEC, for 100s averaging time, is 500/15=33. Considering the length factor of 4.67/0.74 = 6.3, the remaining 33/6.3=5.3 times NEC deterioration is believed to be caused by the non-optimized alignment between the SR-AR-HCF and the pump input SMF, which could result in different power loss for the pump at the joint as well as different fractional pump power η in the LP_{01} mode. For experiments with both HCF samples, the pump power from the input SMF is ~272 mW. We estimated that the pump power into the 4.67-m-long SR-AR-HCF by deducting a joint loss of 4 dB, giving a value of 108 mW. For the 0.74-m-long sample, the joint loss could be larger and the pump power into the HCF smaller.

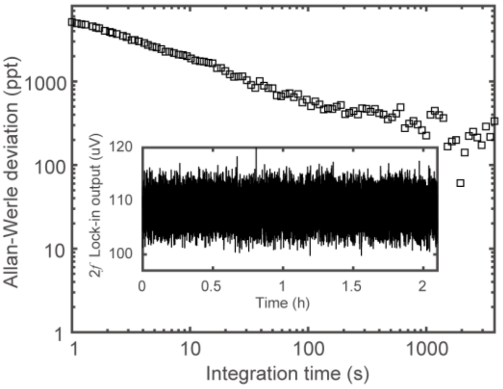

Supplementary Figure 9 Allan-Werle deviation of the 0.74-m-long SR-AR-HCF. The analysis is based on the noise data over a period of 2 hours, which is shown in the inset. The gas pressure is ~ 1.5 bar. The lock-in time constant is 100 ms, corresponding to 0.94 detection bandwidth. The probe power at the PD is about -9.5 dBm.

Supplementary Note 6: Non-linear response of the system

The non-linearity of the system response could be affected by two factors: the non-linear characteristic of the Beer-Lambert law and the non-linear (cosine) transfer function of interferometric detection.

According to the Beer-Lambert law, the differential PT phase modulation can be expressed as

$$\delta\phi = k^*(1\text{-}e^{-\alpha CL})P_{\text{pump}} \approx k^*\alpha CLP_{\text{pump}} \quad (6.1)$$

The above approximation is only accurate in the weak absorption limit, i.e., $\alpha CL << 1$. The relative error from linear approximation is

$$\epsilon_1 = \frac{(1-e^{-\alpha CL})-\alpha CL}{\alpha CL} \times 100\% \quad (6.2)$$

Obviously ϵ_1 will become larger for increasing αCL. For the P(13) line of acetylene with $\alpha = 1.0513\ \text{cm}^{-1}$, concentration C = 1% and SR-AR-HCF length L = 0.74 m, $1 - e^{-\alpha CL} \approx 50\%$ meaning about half of the pump power is absorbed by the gas, and the relative error ϵ_1 is ~30%.

Around the quadrature point, the probe intensity modulation at the output of the dual-mode interferometer is related to the differential PT phase modulation by

$$I_{signal} = \text{A}\, sin(\delta\phi) \approx \text{A}\delta\phi \propto C \quad (6.3)$$

where A is a constant related to the probe power level and the fringe contrast. The approximation is accurate for $\delta\phi$<<1 because of $\sin(\delta\phi) \approx \delta\phi$. The relative error from linear approximation is

$$\epsilon_2 = \frac{\sin(\delta\phi)-\delta\phi}{\delta\phi} \times 100\% \quad (6.4)$$

For larger $\delta\phi$, the relative error ϵ_2 will become larger. With the same parameters above and for 110mW pump power, the differential PT phase modulation is

$$\delta\phi = k^*\alpha CLP_{\text{pump}} = 1.6 \times 10^{-7} \times 1.0513 \times \left(\frac{1\%}{1\text{ppm}}\right) \times 0.74 \times 110 = 0.137 \text{ rad}$$

The relative error ϵ_2 is ~0.3%, well below the relative error ϵ_1. Hence it does not need to be considered here.

Supplementary References:

[1] Arndt R. Analytical line shapes for Lorentzian signals broadened by modulation. *J. Appl. Phys.* **36**, 2522-2524 (1965).

[2] Snyder, A. W. & Love, J. Optical waveguide theory. (Springer Science & Business Media , New York, 2012)

[3] Davis, C. C. & Petuchowski, S. J. Phase fluctuation optical heterodyne spectroscopy of gases. *Appl. optics* **20**, 2539-2554 (1981).

[4] Fokoua, E. N. et al. How to make the propagation time through an optical fiber fully insensitive to temperature variations. *Optica* **4**, 659-668 (2017).

[5] Dangui, V., Kim, H. K., Digonnet, M. J. & Kino, G. S. Phase sensitivity to temperature of the fundamental mode in air-guiding photonic-bandgap fibers. *Opt. express* **13**, 6669-6684 (2005).

[6] Pang, M., Xuan, H. F., Ju, J. & Jin, W. Influence of strain and pressure to the effective refractive index of the fundamental mode of hollow-core photonic bandgap fibers. *Opt. express* **18**, 14041-14055 (2010).

[7] Osório, J. H. et al. Tailoring modal properties of inhibited-coupling guiding fibers by cladding modification. *Sci. Rep.* **9**, 1376 (2019).

[8] Hoo Y. L. et al. Design and modeling of a photonic crystal fiber gas sensor. *Appl. Optics* **42**,

3509-3515 (2003).
[9] Suda, A. et al. Generation of sub-10-fs, 5-mJ-optical pulses using a hollow fiber with a pressure gradient. *Appl. Phys. Lett.* **86**, 111-116 (2005).
[10] Swann, W. C. & Gilbert. S. L. Pressure-induced shift and broadening of 1510–1540-nm acetylene wavelength calibration lines. *J. Opt. Soc. Am. B* **17**, 1263-1270 (2000).
[11] Howe D. A., Allan D. U. & Barnes J. A. Properties of signal sources and measurement methods. *Proc. 35th Ann. Freq. Control Symposium* 669-716 (1981).
[12] Gao, S. F., Wang, Y. Y., Liu, X. L., Ding, W., & Wang, P. Bending loss characterization in nodeless hollow-core anti-resonant fiber. *Opt. Express* **24**, 14801-14811 (2016).
